# Peripheral mechanisms contribute to comorbid visceral hypersensitivity induced by preexisting orofacial pain and stress in female rats

**DOI:** 10.1111/nmo.13833

**Published:** 2020-03-10

**Authors:** Yaping Ji, Bo Hu, Charles Klontz, Jiyun Li, Dean Dessem, Susan G. Dorsey, Richard J. Traub

**Affiliations:** ^1^ Department of Neural and Pain Sciences School of Dentistry University of Maryland Baltimore Baltimore MD USA; ^2^ UM Center to Advance Chronic Pain Research University of Maryland Baltimore Baltimore MD USA; ^3^ Department of Pain and Translational Symptom Science School of Nursing University of Maryland Baltimore Baltimore MD USA; ^4^ Present address: Key laboratory of Shaanxi Province for Craniofacial Precision Medicine Research Xi’an Jiao Tong University College of Stomatology Xi’an Shaanxi China

**Keywords:** astressin, colon, corticotrophin‐releasing factor, Mast cell, resilient, susceptible, visceromotor response

## Abstract

**Background:**

Stress exacerbates many chronic pain syndromes including irritable bowel syndrome (IBS). Among these patient populations, many suffer from comorbid or chronic overlapping pain conditions and are predominantly female. Nevertheless, basic studies investigating chronic psychological stress‐induced changes in pain sensitivity have been mostly carried out in male rodents. Our laboratory developed a model of comorbid pain hypersensitivity (CPH) (stress in the presence of preexisting orofacial pain inducing chronic visceral pain hypersensitivity that significantly outlasts transient stress‐induced pain hypersensitivity (SIH)) facilitating the study of pain associated with IBS. Since CPH and SIH are phenotypically similar until SIH resolves and CPH persists, it is unclear if underlying mechanisms are similar.

**Methods:**

In the present study, the visceromotor response (VMR) to colorectal distention was recorded in the SIH and CPH models in intact females and ovariectomized rats plus estradiol replacement (OVx + E2). Over several months, rats were determined to be susceptible or resilient to stress and the role of peripheral corticotrophin‐releasing factor (CRF) underlying in the pain hypersensitivity was examined.

**Key Results:**

Stress alone induced transient (3‐4 weeks) visceral hypersensitivity, though some rats were resilient. Comorbid conditions increased susceptibility to stress prolonging hypersensitivity beyond 13 weeks. Both models had robust peripheral components; hypersensitivity was attenuated by the CRF receptor antagonist astressin and the mast cell stabilizer disodium cromoglycate (DSCG). However, DSCG was less effective in the CPH model compared to the SIH model.

**Conclusions and Inferences:**

The data indicate many similarities but some differences in mechanisms contributing to comorbid pain conditions compared to transient stress‐induced pain.

## Graphical abstract

Peripheral mechanisms underlying comorbid IBS‐like pain are qualitatively similar to stress‐induced pain during the transient phase of visceral hypersensitivity in female rats. Treating patients for IBS comorbid with other overlapping pain conditions might be similar to IBS in the absence of comorbid pain.

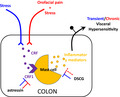

**Key Points**

- Stress in the presence of preexisting orofacial pain induces de novo chronic visceral hypersensitivity that outlasts visceral hypersensitivity induced by stress alone.
- Not all animals are susceptible to stress; some do not become hypersensitive, they are resilient.
- Similar peripheral mechanisms underlie visceral hypersensitivity in the stress‐induced and comorbid pain models.

## 1 INTRODUCTION

Irritable bowel syndrome (IBS) is one of the most common gastrointestinal conditions in humans. It is predominant in women and characterized by visceral hypersensitivity and pain. Stress is a significant risk factor in IBS with many patients reporting that prolonged stressful events precede presentation of IBS symptoms or severity of symptoms.^1^, ^2^, ^3^, ^4^ In addition, IBS is often comorbid with one or more chronic pain syndromes that are collectively referred to as Chronic Overlapping Pain Conditions (COPCs).^5^, ^6^, ^7^, ^8^, ^9^ Many of these conditions (eg, temporomandibular disorder (TMD), fibromyalgia, migraine) occur in tissues located distal to and morphologically distinct from the lower gastrointestinal tract, yet IBS‐like symptoms are similar. It is currently unclear, however, if the mechanisms underlying pain hypersensitivity in IBS and IBS comorbid with other pain conditions are the same.

Mechanisms underlying comorbid chronic pain are not well defined. The high comorbidity incidence among chronic pain patients could result from common risk factors including genetic predisposition, sex hormones, and stress. Chronic stress, for example, is well acknowledged to initiate or trigger psychiatric disorders such as anxiety and depression by influencing a wide range of brain areas including the hippocampus, amygdala, and ventromedial prefrontal cortex, all of which are involved in the sensory and emotional perception of pain.^10^, ^11^, ^12^ In animal models, chronic stress induces hypersensitivity in visceral and somatic organs.^13^, ^14^, ^15^, ^16^, ^17^, ^18^, ^19^, ^20^, ^21^, ^22^ Clinical studies also indicate stress induces visceral hypersensitivity. For example, sensory thresholds in response to colorectal distention were lower in IBS patients compared with that of healthy controls, and both groups showed decreased thresholds after mental stress.^23^ In addition, stress‐induced visceral hypersensitivity is sex and hormone dependent. Stress induced longer lasting visceral hypersensitivity in female rats as compared with males,^18^ and both animal studies and clinical investigations suggest that the female sex hormone estrogen facilitates, while male sex hormone testosterone dampens, chronic pain.^18^, ^24^ Despite the well‐accepted knowledge that there is a female predominance in the above‐mentioned chronic pain conditions, studies investigating stress‐induced changes in pain sensitivity have been predominantly carried out in male rodents, and the long‐term (>2 months) effect of stress on visceral pain is understudied.

Corticotrophin‐releasing factor (CRF) in peripheral tissue plays an important role in initiating visceral hypersensitivity. Experiments conducted with colon biopsies from healthy human volunteers indicated that CRF increased colonic mucosal permeability, which was abolished by the CRF antagonist alpha‐helical CRH (9‐41) and the mast cell stabilizer, lodoxamide.^25^ Further, pretreatment with peripherally restricted CRF antagonists in animal studies reduced visceral hypersensitivity induced by stress or a peripherally acting CRF agonist.^26^, ^27^, ^28^, ^29^ However, it is unknown if similar mechanisms contribute to chronic visceral hypersensitivity following orofacial pain and stress. In the current study, peripheral mechanisms underlying stress‐induced and orofacial inflammation + stress‐induced visceral hypersensitivity were examined and compared.

## 2 METHODS

Experiments were performed on cycling adult female Sprague‐Dawley rats (Envigo; 10 weeks old on arrival at the UM School of Dentistry animal facility) or ovariectomized (OVx) rats (Envigo, 9 weeks at time of surgery) with estrogen (E2) replacement. Rats were acclimated to the housing facility at least 7 days prior to entering the study. Rats were not tested for estrous cycle stage to reduce differential stressors to the animals. All protocols were approved by the University of Maryland School of Medicine Institutional Animal Care and Use Committee and conform to the guide for the use of laboratory animals by the International Association for the Study of Pain. This study focused on ovariectomized and intact female rats as we have shown that the currently used stress paradigms resulted in significantly shorter duration visceral hypersensitivity in both the stress and comorbid pain models in male SD rats (RJ Traub, S Hanson, Y Xue, unpublished observations).^18^

### 2.1 Surgery

Rats were anesthetized with isoflurane (5% for induction, 2% for maintenance), and electromyogram (EMG) electrodes made from 40 AWG 10/50 stranded stainless steel wire (AS631; Cooner Wire Co) were implanted in the abdominal muscle 10 days prior to recording. Rats were subsequently single housed to avoid interfering with cagemate's electrodes.

### 2.2 Complete Freund's Adjuvant injection in masseter muscle

One day prior to starting the stress protocol, rats were briefly sedated with isoflurane, and Complete Freund's Adjuvant (CFA) (Sigma‐Aldrich, F5881; 50 µL, 1:1 in saline) was injected bilaterally into the masseter muscles.

### 2.3 Forced swim stress

Rats were subject to Forced swim (FS) for three successive days.^13^, ^15^, ^30^ Rats were placed in a cylinder (30 cm in diameter, 60 cm in height, filled to 20 cm with tap water, adjusted to a temperature of 26°C). Rats were placed in the water for 10 minutes on the first day and 20 minutes on the following 2 days. After each session, rats were dried in a heated area before being returned to their home cage. FS was carried out at the same time in the morning to avoid any influence of circadian rhythms. The day after the last forced swim was designated day 1.

#### 2.3.1 Restraint stress

Rats were restrained in Broome style rodent restrainers (4.8 cm diameter, 20 cm length) preventing movement for 2 hrs per day for 4 consecutive days.^31^, ^32^ One cohort of rats were left horizontal for the 2‐hours period. Others were tilted at a 45‐degree angle head up or head down in 15‐minutes blocks alternating with 15‐minutes blocks in the horizontal position. The day after the last restraint session was designated day 1.

### 2.4 Visceromotor response

The visceromotor response (VMR) is manifest as changes in the magnitude of the electromyogram (EMG) recorded from the abdominal muscles in response to colorectal distention. The EMG signal was recorded with a CED 1401 and analyzed using Spike 2 for windows software (Cambridge Electronic Design, UK). The EMG was rectified and integrated. Starting 2 days before the baseline recording, rats were acclimated to the rodent restrainers (6.2 cm diameter, 21.5 cm length; larger than the stress restrainers) for 2 hours each day. Rats were then fasted overnight (water ad libitum) to facilitate balloon placement. On the day of the experiment, rats were briefly sedated with isoflurane and a 5‐ to 6‐cm balloon (made from the finger of a surgical glove attached to Tygon tubing) was inserted through the anus into the descending colon and rectum. The distal end of the balloon was maintained 1 cm proximal to the external anal sphincter by taping the tubing to the tail. Rats were then put in the restrainers and allowed 30 minutes to recover from the isoflurane. Three colorectal distention trials were run. Each trial consisted of inflating the distention balloon to 20, 20, 40, 40, 60, 60, 80 and 80 mm Hg (20‐seconds duration, 3‐minutes interstimulus interval). The record was rectified, and the response to each pressure was calculated by subtracting the value for the 20 seconds prior to distention from the value during the 20‐seconds distention. The data are presented as the mean response from the second and third trials on each day. The VMR to colorectal distention was recorded prior to stress (baseline) and 1, 2, 3, 4, 7, 10 and 13 weeks after the last stress session.

### 2.5 Drug/hormone administration

β‐estradiol 3‐benzoate (E2; Sigma‐Aldrich E8515) was dissolved in safflower oil to a final concentration of 0.1 mg mL^−1^. OVx rats were injected with E2 (10 µg) every 4 days. Astressin, a CRF1/2 receptor antagonist that does not cross the blood–brain barrier (Tocris [Cat. No. 1606]), was dissolved in saline to a final concentration of 30 µg mL^−1^. 30 µg kg^−1^ was injected i.p. 30 minutes prior to each FS session. Disodium cromoglycate (DSCG; Sigma‐Aldrich C0399) was dissolved in saline to a final concentration of 50 mg mL^−1^. Rats were injected with 25 or 150 mg kg^−1^, i.p. 30 minutes prior to each restraint stress (RS) session.

### 2.6 Statistics

Data are expressed as mean ± SEM and were analyzed in GraphPad Prism V.6 using *t* test, one‐way or two‐way ANOVA. Bonferroni multiple comparison was used to test between groups as appropriate. *P* < .05 was considered significant. The VMR data are presented two ways. The stimulus–response curves for increasing intensities of colorectal distention were plotted for each time point (day). These data were analyzed by two‐way ANOVA with time and distention pressure as factors. In all cases, there was a significant main effect of pressure (response increased with increasing distention pressure) and it is not reported in the results. If there was a significant effect of time or a time × pressure interaction, time was further analyzed by Bonferroni multiple comparison and reported in the bar graphs showing the area under the curve (AUC) for days poststress. The area under the curve is the sum of the response to the 4 distention pressures on each day.

## 3 RESULTS

### 3.1 Stress‐induced and comorbid pain hypersensitivity

**Figure 1:**
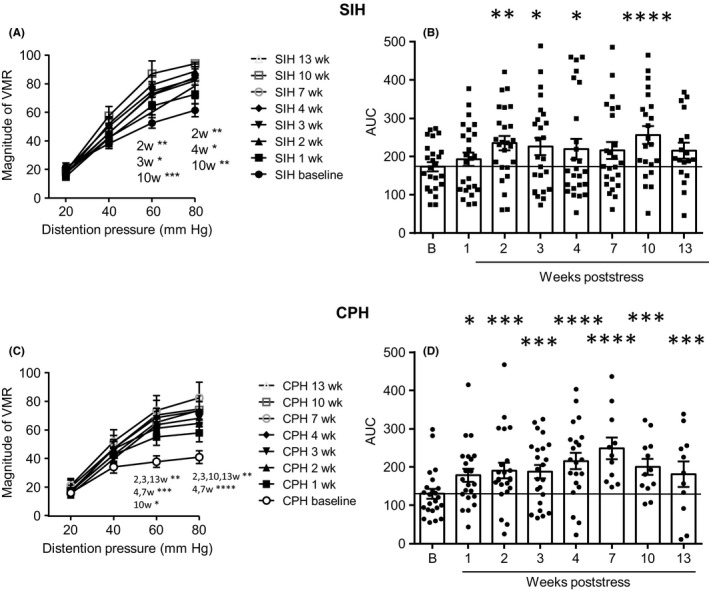
Time course of SIH (A,B) and CPH (C,D). A, C, The VMR at different distention pressures by week. *,**,***,**** *P* < .05, .01, .001, .001 vs baseline at each pressure for the weeks indicated. B, D, The area under the curve for each week reported in panels A and C. Individual animals are indicated by the black symbols. *,**,***,**** *P* < .05, .01, .001, .001 vs baseline

We have reported previously that in ovariectomized rats with E2 replacement, 3 days of FS in the presence of preexisting orofacial pain induced comorbid pain hypersensitivity (CPH) to colorectal distention that persisted significantly longer than visceral hypersensitivity induced by FS alone (stress‐induced hypersensitivity, SIH).^13^ In a separate study, we observed FS induced visceral hypersensitivity in intact female rats that persisted at least 18 days, but <1 week in males.^18^ In the present study, we followed intact female rats for a much longer period (13 weeks). Stress‐induced hypersensitivity (SIH) showed a delayed onset beginning more than 1 week after the cessation of the stressor and persisted between 4 and 7 weeks (2‐way ANOVA, time: *F* (7,732) = 4.6, *P* < .0001; pressure: *F* (3,732) = 166.9, *P* < .0001; n = 25; Figure 1A, B). Visceral sensitivity did not differ over time to lower intensities of colorectal distention (20, 40 mm Hg), but there was a significant increase at higher intensities (60, 80 mm Hg) for several weeks compared to the prestress baseline (Figure 1A). Multiple comparisons showed a significant effect of time compared to baseline (Figure 1B).

In the inflammation plus stress condition, comorbid pain hypersensitivity (CPH) was apparent at 1 week and persisted at least 13 weeks (2‐way ANOVA, time: *F* (7,556) = 6.772, *P* < .0001; pressure: *F* (3,556) = 102.4, *P* < .0001; n = 23; Figure 1C,D). Similar to stress alone, there was an increase in visceral sensitivity at higher distention pressures (60 and 80 mm Hg), but not at lower pressures. As a control, injection of CFA without stress failed to elicit visceral hypersensitivity in intact females over 13 weeks (RM ANOVA, treatment: *F* (3,459, 17.30) = 2.735, *P* = .0690). This was similar to what we previously reported in OVx + E2 replacement rats.^13^

**Figure 2:**
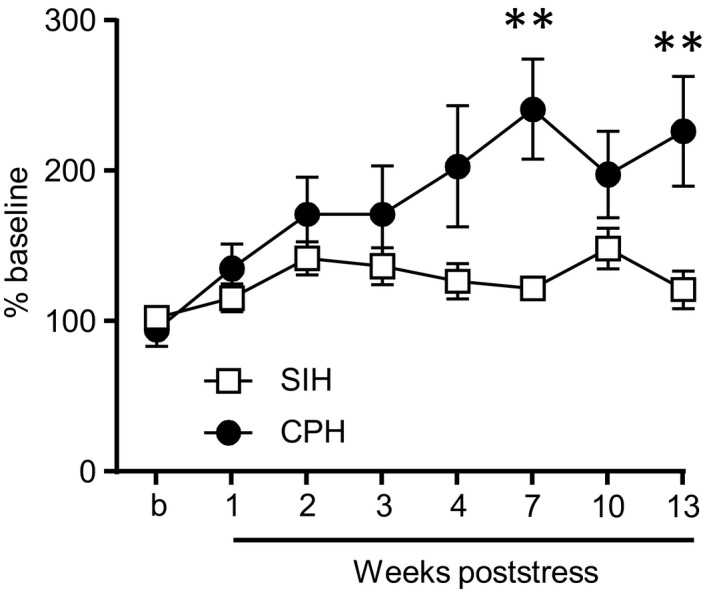
The magnitude of the VMR (area under the curve) normalized to baseline for stressed and comorbid rats. ** *P* < .01 vs the same time in stressed (SIH) rats

There was no difference in the magnitude of the visceral hypersensitivity between CPH and SIH groups over the first 4 weeks poststress, but overall there was a significant difference over the 13 weeks (2‐way ANOVA, time × treatment: *F* (7,316) = 2.710, *P* = .0097; time: *F* (7,316) = 4.371, *P* = .0001; treatment: *F* (1,316) = 19.44, *P* < .0001; Figure 2).

Further examination of the responses following stress suggested some rats did not exhibit visceral hypersensitivity in both the SIH and CPH groups (Figure 1B,D). This is consistent with reports by others showing not all rats are susceptible to stress (ie, some rats are resilient), which mirrors what some studies show in humans.^33^, ^34^, ^35^ We examined the intact female SIH and CPH rats for this characteristic. Rats were parsed based on the magnitude of hypersensitivity observed 1 week following the cessation of the stress paradigm. Rats were separated into susceptible to stress (the magnitude of the VMR following stress was greater than baseline) or resilient (the magnitude was less than or equal to baseline).

**Figure 3:**
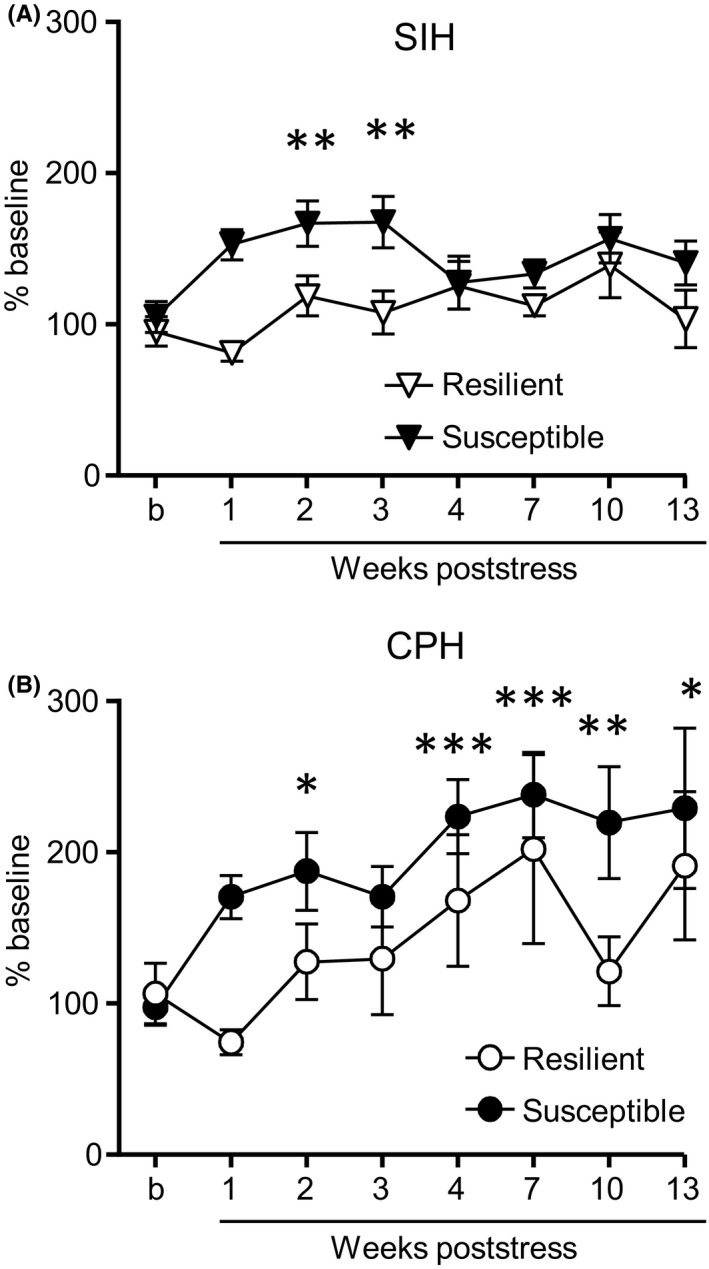
Separation of SIH (A) and CPH (B) rats into susceptible (closed symbol) and resilient (open symbol) groups. The response of resilient rats was never significantly greater than baseline. *,**,*** *P* < .05, .01, .001 vs baseline

In SIH rats, half the rats were susceptible to stress (12/25) and the remaining rats were resilient. The mean response at 1 week was significantly greater in the susceptible group (152.8% of baseline, range 110%‐235%) compared to the resilient group (81.4% of baseline, range 49%‐100%; *t* test: *t* = 6.297, *df* = 23, *P* < .0001). Over the 13 weeks, the response of rats susceptible to stress was significantly greater than those resilient to stress (2‐way ANOVA; time: *F* (7,171) = 2.569, *P* = .0153; treatment: *F* (1,171) = 23.09, *P* < .0001; Figure 3A, Figure S1A). Susceptible rats had visceral hypersensitivity through 3 weeks before resolving (1‐way ANOVA; *F* (7,82) = 2.410, *P* = .0270). There was no hypersensitivity in rats resilient to stress.

A similar analysis was used in the comorbid rats; 74% (17/23) were susceptible to stress, while the remaining 6 were resilient. The mean response at 1 week was 170.6% ± 14.4% of baseline (range: 114%‐327%) for the rats susceptible to stress vs 74.3% ± 8.2% (range 47%‐100%) for the resilient rats (*t* test: *t* = 3.839, *df* = 21, *P* = .0010). Over the 3 months that data were collected, the response of susceptible comorbid rats was significantly greater than resilient comorbid rats (2‐way ANOVA; time: *F* (7,129) = 3.763, *P *= .0010; treatment: *F* (1,129) = 11.29, *P* = .0010; Figure 3 B, Figure S1B). In the susceptible rats, there was a significant increase in the VMR at most time points compared to baseline (1‐way ANOVA; *F* (7,94) = 4.271, *P* = .0004). In contrast, there was no hypersensitivity at any timepoint in the resilient rats (1‐way ANOVA; *F* (7,35) = 1.500, *P* = .1995).

Although data on the estrous cycle stage were not collected in this study, we tested if being resilient or susceptible to stress was correlated with E2 status by reanalyzing data from our previous publication.^13^ In ovariectomized rats with E2 replacement, 93% (13/14) of comorbid rats were susceptible to stress. In contrast, only 14% (1/7) ovariectomized rats treated with orofacial inflammation plus stress developed visceral hypersensitivity, suggesting E2 increased the effect of stress on visceral sensitivity.^18^

**Figure 4:**
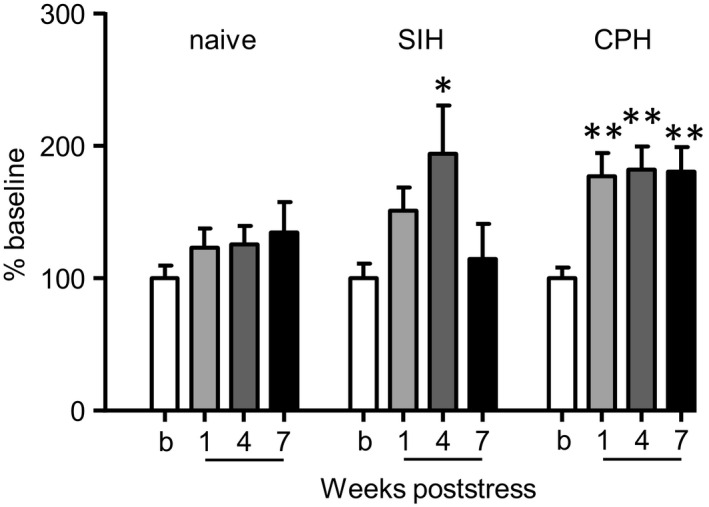
The effects of restraint stress on the response to colorectal distention in SIH and CPH rats normalized to the baseline response. *,** *P* < .05, 0.01 vs. baseline (b). N = 10‐13/group

In order to confirm the chronic vs transient effects of stress on visceral sensitivity, a second stressor (4 days of RS) was tested. Restraint stress induced visceral hypersensitivity in both SIH (1‐way ANOVA; *F* (3,36) = 2.919, *P* = .0472; n = 10) and CPH (1‐way ANOVA; *F* (3,36) = 6.199, *P* = .0017; n = 10) rats (Figure 4). The SIH rats were hypersensitive to visceral stimulation at 4 weeks, but the hypersensitivity resolved by 7 weeks. In the CPH group, rats were hypersensitive for at least 7 weeks. When the susceptible/resilient algorithm was applied, there were no rats resilient to stress in the CPH group and only 20% were resilient in the SIH group.

Interestingly, restraint alone (keeping rats in tubes for 2 h/d for 4 days in a horizontal position) did not induce visceral hypersensitivity. The hypersensitivity only developed when the tubes were placed at a 45° angle head up or head down alternating with horizontal placement in 15‐minutes increments for 2 h/d for 4 days (Figure S2).

Overall, independent of stressor, significantly more comorbid rats (CPH, 82%) were susceptible to stress developing visceral hypersensitivity compared to stress only rats (SIH, 57%; chi‐square, *P* = .0368), suggesting preexisting pain (muscle inflammation) increases susceptibility to stress when measuring visceral sensitivity. Furthermore, restraint as a stressor (CPH and SIH combined) resulted in a greater percentage of rats classified as susceptible (90%) compared to the forced swim stress (60%), suggesting restraint might be a more potent stressor (chi‐square *P* = .0207).

There was no difference in the baseline response to colorectal distention between the SIH and CPH rats that were determined to be susceptible or resilient to stress, suggesting poststress behavior cannot be predicted from the baseline VMR recording (Figure S3).

### 3.2 Peripheral corticotrophin‐releasing factor

**Figure 5:**
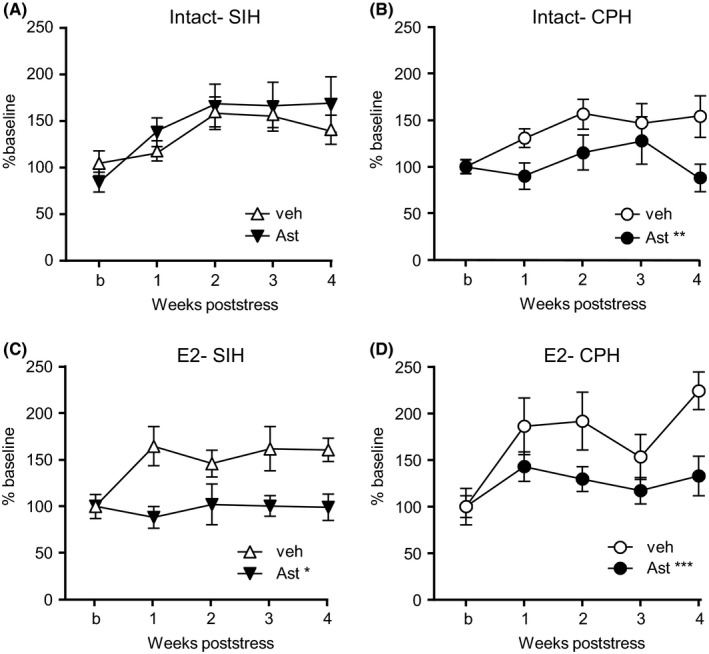
The effect of systemic astressin on SIH and CPH in intact (A,B) and ovariectomized + E2 (C,D) female rats. *,**,*** *P* < .05, .01, .001 vs vehicle

Stress evokes the release of CRF in the colon that contributes to visceral hypersensitivity.^27^, ^29^, ^36^, ^37^, ^38^ In intact rats, pretreatment with astressin, the peripherally restricted, nonselective CRF receptor antagonist, blocked the development of SIH (1‐way ANOVA: *F* (4,48) = 1.743, *P* = .1559). However, the vehicle‐treated rats did not develop visceral hypersensitivity either, and therefore, there was no difference between pretreatment with astressin and vehicle in the SIH rats (2‐way ANOVA: treatment, *F* (1,103) = 0.4976, *P* = .4822; Figure 5A). The most likely explanation is that many of the vehicle‐treated rats would have been resilient to stress reducing the overall response to distention. Astressin also blocked the development of CPH, which developed in the vehicle‐treated rats (2‐way ANOVA; treatment, *F* (1,79) = 4.600, *P* = .0350; Figure 5B). In contrast, astressin administered for 3 days following establishment of CPH (injected on days 12‐14 poststress) had no effect on visceral hypersensitivity (1‐way RM ANOVA, *F* (2.096,20.96) = 7.636, *P* = .0029; Figure S4). This suggests that targeting the CRF signaling system may not be therapeutic in patients that already have IBS.

The effect of astressin was also tested in ovariectomized rats with estrogen replacement. Consistent with the results from intact female rats, in both the stressed and comorbid rats, 30 µg kg^−1^ astressin prevented the development of visceral hypersensitivity compared to vehicle (2‐way ANOVA; SIH: treatment, *F* (1,55) = 22.53, *P* < .0001; CPH: time, *F* (4,51) = 4.514, *P* = .0034; treatment, *F* (1,51) = 12.50, *P* = .0009; Figure 5C,D).

**Figure 6:**
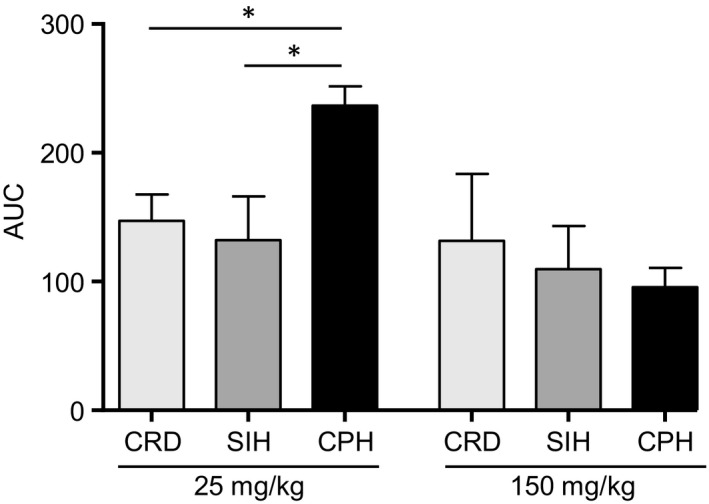
Effects of DSCG on SIH and CPH in intact female rats. * *P* < .05. CRD: distention without restraint stress

Although astressin is not supposed to cross the blood–brain barrier^39^ and therefore act in the periphery, we confirmed that visceral hypersensitivity in SIH and CPH rats resulted from a peripheral action of stress. The effect of disodium cromoglycate (DSCG; 25 and 150 mg kg^−1^, s.c.), a mast cell stabilizer that prevents degranulation, was tested as a pretreatment. DSCG (25 mg kg^−1^) blocked emergence of SIH, but not CPH (1‐way ANOVA, *F* (2,10) = 6.261, *P* = .0173; Figure 6). In contrast, 150 mg kg^−1^ DSCG blocked the development of both SIH and CPH (1‐way ANOVA; *F* (2,11) = 0.3155, *P* = .7358).

## 4 DISCUSSION

In the current study, we examined mechanisms underlying pain associated with COPCs and ask if the underlying cause of the chronic visceral hypersensitivity following stress in rats with preexisting orofacial pain is mechanistically similar to the transient visceral hypersensitivity induced by stress in healthy female rats.^19^, ^40^, ^41^ Our results indicate that the same stressor that induces transient visceral hypersensitivity induces chronic visceral hypersensitivity when the rat has a preexisting orofacial pain condition. Additionally, the data suggest that the intensity of the stressor and hormonal conditions act together to increase the intensity of visceral hypersensitivity. In this report, we further suggest that peripheral mechanisms that contribute to SIH are also involved in the comorbid pain model.

### 4.1 Stress and visceral pain

The effects of intense or chronic stress on visceral or orofacial pain have been extensively examined in basic and preclinical animal studies and reported to increase pain (in contrast to acute stress‐induced analgesia).^16^, ^19^, ^20^, ^42^, ^43^, ^44^, ^45^, ^46^ However, the effect of stress on pain sensitivity was only examined in otherwise healthy individuals or in previously injured tissue. For example, forced swim stress in Sprague‐Dawley (SD) rats induced thermal and chemical hyperalgesia that persisted 8‐9 days 30, 47 and exacerbated orofacial pain.^45^, ^48^ Stress increased visceral sensitivity in SD rats 18, 22, 49 and the high‐anxiety Wistar‐Kyoto rat strain (WKY) exhibited lower thresholds and greater suprathreshold responses to colorectal distention than lower anxiety SD rats in the absence of external stress.^27^, ^33^, ^50^, ^51^ Maternal separation, a model for early life stress, increased visceral and orofacial pain in adult rats which was further exacerbated by an additional stressor in the adult.^52^, ^53^, ^54^, ^55^, ^56^ These studies indicate stress modulates nociceptive processing in otherwise normal individuals. Stress also increased pain in a previously injured limb with a nerve injury.^57^, ^58^ Thus, stress induces de novo visceral hypersensitivity or somatic hyperalgesia in normal individuals or exacerbates pain arising from an injured area. Most importantly, under these conditions, the duration of the stress‐induced hyperalgesia is limited.

In clinical settings, a subset of TMD patients also develop IBS.^5^, ^6^, ^7^ To date, little is known about the mechanisms underlying the effects of stress on visceral pain hypersensitivity in the presence of an unrelated preexisting orofacial pain condition, modeling patients with TMD who develop IBS. It is acknowledged that stress is a significant risk factor for developing or exacerbating chronic disease states including many COPCs. Notably patients diagnosed with IBS report periods of prolonged or intense stress precede presentation of their symptoms.^19^, ^40^, ^41^ In addition, most chronic pain conditions are more prevalent in or exclusive to women.^9^, ^59^, ^60^, ^61^, ^62^ Yet historically, most basic science studies were conducted in male rodents although as NIH has called for the inclusion of sex as a biological variable, more recent studies include females.^18^, ^21^, ^27^, ^33^, ^44^, ^63^, ^64^, ^65^, ^66^, ^67^ Generally, these studies show stress induces transient visceral hypersensitivity. In most cases, it is measured within one day of the cessation of the stress stimulus, but has been reported to persist one month in male Wistar rats.^33^ The current study establishes a model in the female rat by showing that the same stressor that induces transient visceral hypersensitivity (3‐4 weeks) evokes long‐lasting (>13 weeks) visceral hypersensitivity in the presence of a preexisting orofacial pain.

A key aspect of the effects of stress on pain is the extent to which an individual is resilient. Stress is an aspect of everyday life, yet only a portion of individuals are adversely affected. Chronic stress has more profound effects on susceptible individuals contributing to adverse outcomes (see Ref. 19, 68 for review). In our animal studies, 3 days of forced swim stress resulted in transient (~4 weeks) visceral hypersensitivity in approximately half the females tested. However, in males, this same stressor induced hypersensitivity of much shorter duration.^18^ Using a different stressor, 4 days of RS, 80% of the intact females were hypersensitive between 4 and 7 weeks. For comparison, a longer, possibly even more robust stressor, 10 days of water avoidance stress, resulted in visceral hypersensitivity in 70% of male Wistar rats, some remaining hypersensitive for 1 month; females were not examined.^33^ In addition, estrogen modulated the response to stress under the same conditions as when E2 increased visceral sensitivity in OVx rats.^69^ Therefore, when E2 was at a high level following administration in OVx rats, the majority of rats were susceptible. It is unclear, however, if E2 directly modulates CRF circuitry in the periphery or if there is a central interaction between the effects of stress and the effects of E2. Nonetheless, these data suggest that the intensity of the stressor can strongly affect the intensity and duration of the pathological outcome. Further, our data show that gonadal hormones have a significant effect on the consequences of stress.

### 4.2 Role of peripheral CRF in SIH and CPH

In the first several weeks, the magnitude of visceral hypersensitivity was similar in the SIH and CPH models. One possibility is this was due to a ceiling effect in the magnitude of the VMR. Alternatively, it can be hypothesized that similar mechanisms underlie the increase in visceral hypersensitivity in the SIH and CPH models. In this report, we specifically addressed whether peripheral mechanisms that are known to contribute to SIH are similarly involved in the comorbid pain model. This was tested by using the peripherally restricted CRF1/2 receptor antagonist astressin and the mast cell stabilizer DSCG.

The role of peripheral CRF and CRF receptors (CRF1/2) in the colon has been extensively reviewed.^37^, ^70^, ^71^, ^72^, ^73^, ^74^ Stress increases visceral sensitivity, in part by elevating CRF and the CRF‐related peptides urocortin (Ucn) 1, 2, and 3 in the GI tract. Peripheral CRF is released from sensory, sympathetic and enteric neurons, immune cells, and epithelial cells and binds CRF1 receptors activating colonic myenteric neurons and degranulating mast cells.^37^, ^75^, ^76^, ^77^, ^78^ CRF1 and CRF1/2 antagonists block stress‐induced visceral hypersensitivity. This suggests CRF or Ucn 1, the ligands for CRF1, contributes to colonic sensation. Peripheral injection of CRF or CRF1 agonists mimics stress increasing epithelial permeability, colonic motility, GI transit, defecation, and diarrhea.^26^, ^37^, ^74^, ^79^ In contrast, selective activation of CRF2 acts as a negative modulator decreasing mast cell degranulation and inhibiting visceral sensitivity.^71^, ^80^, ^81^ In addition, CRF increases release of inflammatory mediators in the gut wall that induces peripheral sensitization increasing visceral sensitivity.^70^, ^82^, ^83^ Similar to previous studies, astressin blocked the development of SIH in both intact females and ovariectomized rats treated with E2 replacement compared to controls although the specific effect of CRF or Ucn1 was not determined. We now show that pretreatment with astressin blocked the development of visceral hypersensitivity in the comorbid pain model in both ovariectomized and intact female rats.

Mast cells express CRF1 and 2 receptors.^25^, ^77^ During the response to stress, peripheral CRF binds CRF1 receptors degranulating colonic mast cells releasing inflammatory mediators (eg, histamine, 5‐HT, TNF‐α, proteases) that directly or indirectly sensitize colonic sensory afferent fibers.^83^, ^84^, ^85^, ^86^, ^87^ In contrast, activation of CRF2 inhibits mast cell degranulation.^88^ Mast cell degranulation increased visceral sensitivity and inhibiting mast cell degranulation attenuated stress‐induced visceral hypersensitivity in rats^89^ (van den Wijngaard, 2012 #8086; Carroll, 2013 #8751) and is useful clinically for stress associated IBS symptoms.^90^, ^91^ In the current study, we tested the effect of two doses of the mast cell stabilizer DSCG on stress‐induced visceral hypersensitivity in intact female rats. Our results indicate low dose (25mg kg^−1^) DSCG attenuated SIH, but had no effect in CPH while high dose (150 mg kg^−1^) attenuated visceral hypersensitivity in both models. Interestingly, a previous study showed 25 mg kg^−1^ was not effective in Wistar‐Kyoto rats, a strain that has lower thresholds to distention compared to Sprague‐Dawley rats.^50^ Collectively, these data suggest that more robust stress conditions produce a stronger MC response.

## 5 CONCLUSION

The current study shows that experiencing prolonged or chronic stress while in an existing pain state prolongs the effects of stress on visceral hypersensitivity. The strength of the stressor and estrogen status affects the resiliency of the individual to develop transient or chronic pain. The magnitude of pain hypersensitivity is similar for stress‐induced and comorbid pain, and contributing peripheral mechanisms are qualitatively similar, but quantitatively different. The differences may reflect additional factors not examined at this time.

## CONFLICT OF INTEREST

The authors confirm they have no competing interests.

## AUTHOR CONTRIBUTIONS

YJ, BH, CK, JL performed the research; RJT, SGD, and DD designed the research study; YJ, BH, and RJT analyzed the data; YJ and RJT wrote the paper.

## Supporting information

Fig S1‐S4
