# Supplementary material for: Peripheral mechanisms contribute to comorbid visceral hypersensitivity induced by preexisting orofacial pain and stress in female rats
Source: Neurogastroenterol Motil. 2020 Mar 10;32(7):e13833. doi: 10.1111/nmo.13833 (PMC7319894; doi:10.1111/nmo.13833)
Supplement: Supplementary file 1 — Fig S1‐S4 [file NMO-32-e13833-s001.pptx]

## Slide 1
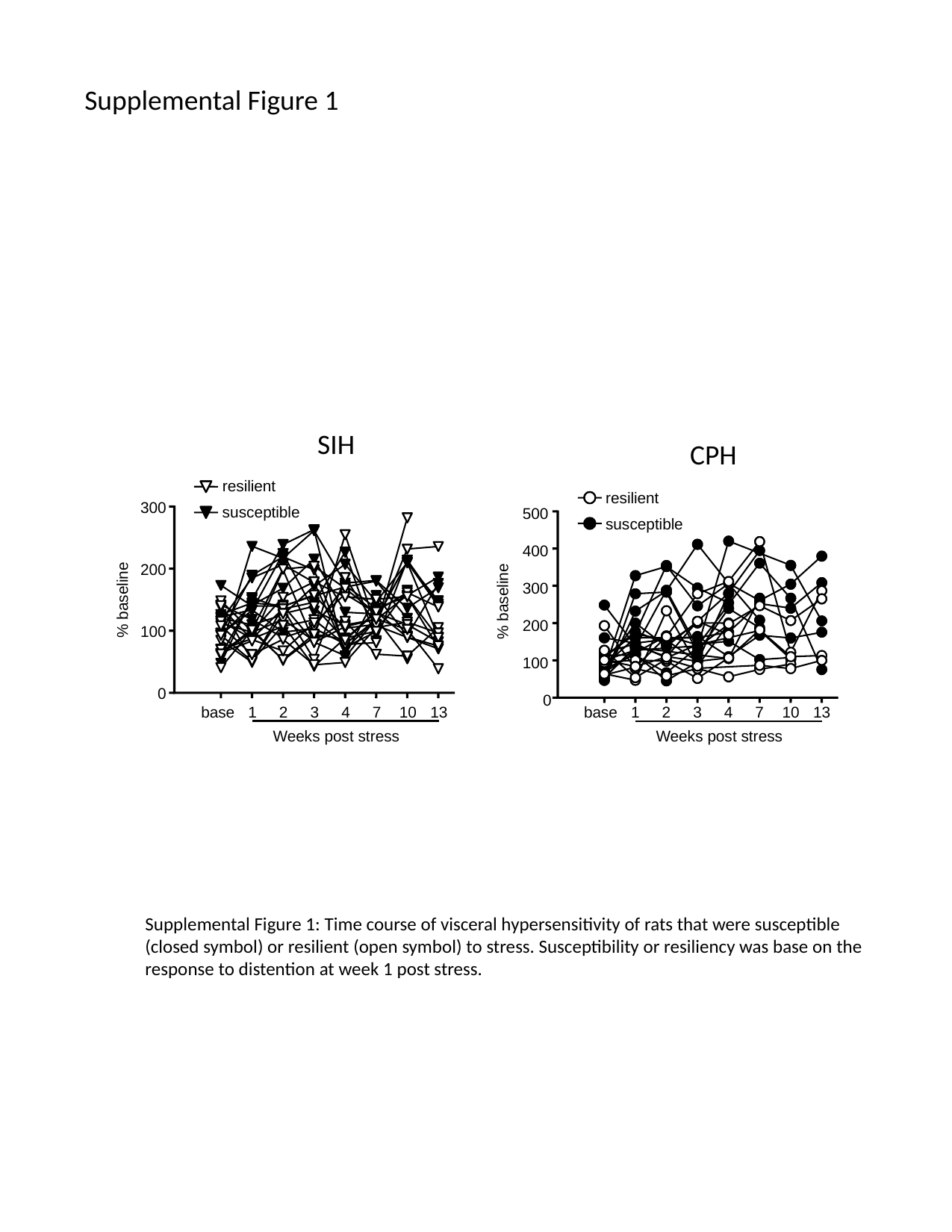

Supplemental Figure 1
SIH
resilient
300
susceptible
200
% baseline
100
0
base
1
2
3
4
7
10
13
Weeks post stress
CPH
resilient
500
susceptible
400
300
% baseline
200
100
0
base
1
2
3
4
7
10
13
Weeks post stress
Supplemental Figure 1: Time course of visceral hypersensitivity of rats that were susceptible (closed symbol) or resilient (open symbol) to stress. Susceptibility or resiliency was base on the response to distention at week 1 post stress.

## Slide 2
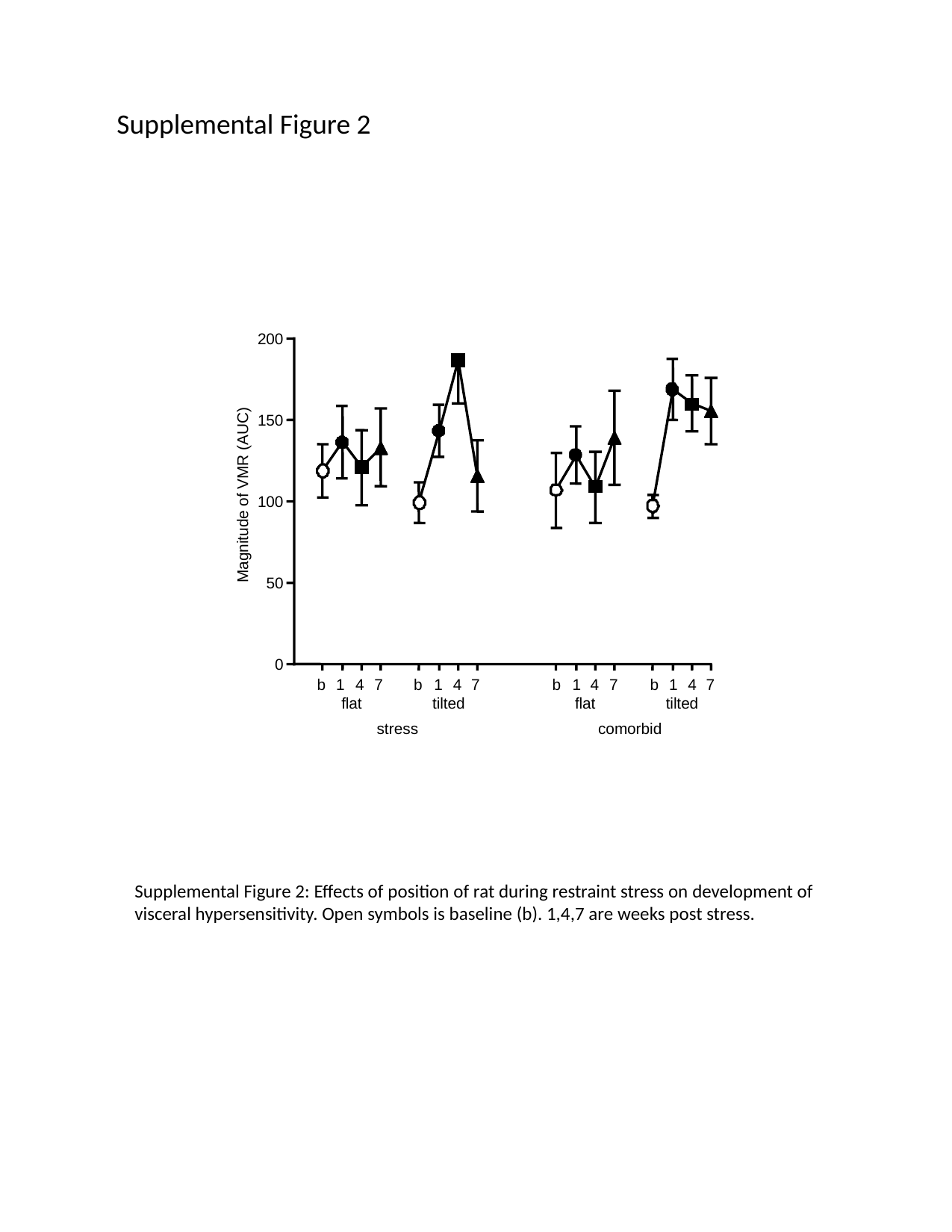

Supplemental Figure 2
200
b
1
4
7
tilted
b
1
4
7
tilted
b
1
4
7
flat
b
1
4
7
flat
150
Magnitude of VMR (AUC)
100
50
0
stress
comorbid
Supplemental Figure 2: Effects of position of rat during restraint stress on development of visceral hypersensitivity. Open symbols is baseline (b). 1,4,7 are weeks post stress.

## Slide 3
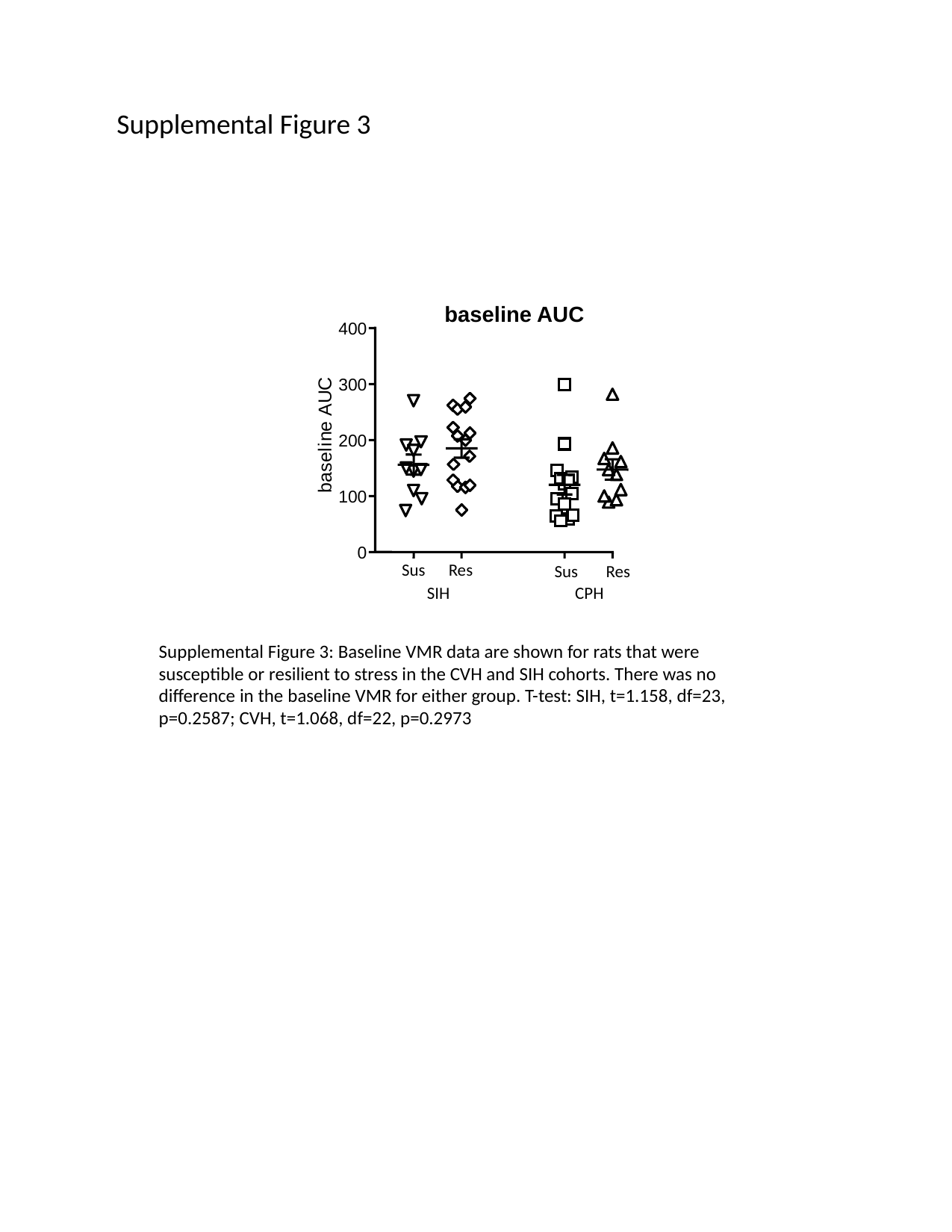

Supplemental Figure 3
baseline AUC
400
C
300
U
A
e
n
200
i
l
e
s
a
b
100
0
Sus
Res
CPH
Sus
Res
SIH
Supplemental Figure 3: Baseline VMR data are shown for rats that were susceptible or resilient to stress in the CVH and SIH cohorts. There was no difference in the baseline VMR for either group. T-test: SIH, t=1.158, df=23, p=0.2587; CVH, t=1.068, df=22, p=0.2973

## Slide 4
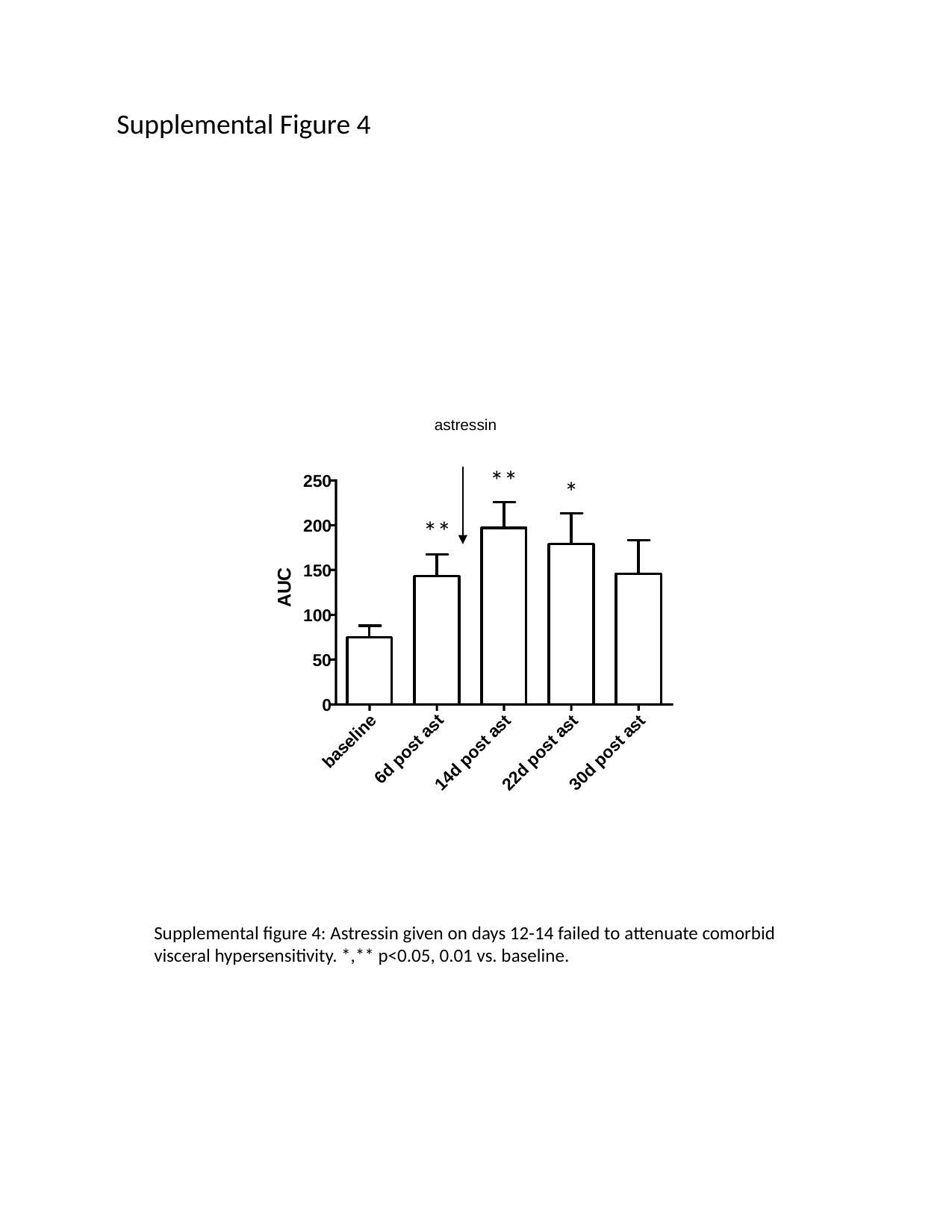

Supplemental Figure 4
astressin
**
*
250
**
200
150
C
U
A
100
50
0
t
t
t
t
e
s
s
s
s
n
a
a
a
a
i
l
t
t
t
t
e
s
s
s
s
s
o
o
o
o
a
p
p
p
p
b
d
d
d
d
6
4
2
0
1
2
3
Supplemental figure 4: Astressin given on days 12-14 failed to attenuate comorbid visceral hypersensitivity. *,** p<0.05, 0.01 vs. baseline.
